# The anticonvulsant phytocannabinoids CBGVA and CBDVA inhibit recombinant T-type channels

**DOI:** 10.3389/fphar.2022.1048259

**Published:** 2022-11-01

**Authors:** Michael Udoh, Chris Bladen, Marika Heblinski, Jia Lin Luo, Vaishali S. Janve, Lyndsey L. Anderson, Iain S. McGregor, Jonathon C. Arnold

**Affiliations:** ^1^ Lambert Initiative for Cannabinoid Therapeutics, The University of Sydney, Sydney, NSW, Australia; ^2^ Discipline of Pharmacology, Sydney Pharmacy School, Faculty of Medicine and Health, The University of Sydney, Sydney, NSW, Australia; ^3^ Brain and Mind Centre, The University of Sydney, Sydney, NSW, Australia; ^4^ Macquarie Medical School, Macquarie University, Sydney, NSW, Australia; ^5^ School of Psychology, Faculty of Science, The University of Sydney, Sydney, NSW, Australia

**Keywords:** CBDVA, CBGVA, Cav3.1, Cav3.2, GPR55, Phytocannabinoids

## Abstract

**Introduction:** Cannabidiol (CBD) has been clinically approved for intractable epilepsies, offering hope that novel anticonvulsants in the phytocannabinoid class might be developed. Looking beyond CBD, we have recently reported that a series of biosynthetic precursor molecules found in cannabis display anticonvulsant properties. However, information on the pharmacological activities of these compounds on CNS drug targets is limited. The current study aimed to fill this knowledge gap by investigating whether anticonvulsant phytocannabinoids affect T-type calcium channels, which are known to modulate neuronal excitability, and may be relevant to the anti-seizure effects of this class of compounds.

**Materials and methods:** A fluorescence-based assay was used to screen the ability of the phytocannabinoids to inhibit human T-type calcium channels overexpressed in HEK-293 cells. A subset of compounds was further examined using patch-clamp electrophysiology. Alphascreen technology was used to characterise selected compounds against G-protein coupled-receptor 55 (GPR55) overexpressed in HEK-293 cells, as GPR55 is another target of the phytocannabinoids.

**Results:** A single 10 µM concentration screen in the fluorescence-based assay showed that phytocannabinoids inhibited T-type channels with substantial effects on Ca_v_3.1 and Ca_v_3.2 channels compared to the Ca_v_3.3 channel. The anticonvulsant phytocannabinoids cannabigerovarinic acid (CBGVA) and cannabidivarinic acid (CBDVA) had the greatest magnitudes of effect (≥80% inhibition against Ca_v_3.1 and Ca_v_3.2), so were fully characterized in concentration-response studies. CBGVA and CBDVA had IC_50_ values of 6 μM and 2 µM on Ca_v_3.1 channels; 2 μM and 11 µM on Ca_v_3.2 channels, respectively. Biophysical studies at Ca_v_3.1 showed that CBGVA caused a hyperpolarisation shift of steady-state inhibition. Both CBGVA and CBDVA had a use-dependent effect and preferentially inhibited Ca_v_3.1 current in a slow inactivated state. CBGVA and CBDVA were also shown to antagonise GPR55.

**Conclusion and implications:** These findings show that CBGVA and CBDVA inhibit T-type calcium channels and GPR55. These compounds should be further investigated to develop novel therapeutics for treating diseases associated with dysfunctional T-type channel activity.

## 1 Introduction

Plant-derived cannabinoids may provide a new class of anticonvulsant drugs as the phytocannabinoid cannabidiol (CBD, Epidiolex®) was recently approved to treat intractable epilepsies including Dravet syndrome (Marini et al., 2011; Anwar et al., 2019). Cannabis contains over 100 phytocannabinoids, and our laboratory has been investigating whether additional phytocannabinoids display anticonvulsant properties (Anderson et al., 2019; Anderson et al., 2021a; Anderson et al., 2021b).

Using the *Scn1a* ^*+/–*^ mouse model of Dravet syndrome, we have found several plant cannabinoids with novel anti-seizure effects. Interestingly, we have observed a common theme of active compounds being acidic biosynthetic precursor molecules, which are formed *via* enzymatic synthesis in the plant. These acidic phytocannabinoids are abundant in raw cannabis or cold-extracted products and undergo decarboxylation when cannabis is heated. Notably, we found cannabichromenic acid (CBCA), cannabichrovarinic acid (CBCVA), cannabigerolic acid (CBGA), cannabigerovarinic acid (CBGVA), and cannabidivarinic acid (CBDVA) were all anticonvulsant against hyperthermia-induced seizures in the *Scn1a* ^*+/–*^ mice (Anderson et al., 2019; Anderson et al., 2021a; Anderson et al., 2021b).

There exists a paucity of data on the molecular pharmacology of these acidic phytocannabinoids, particularly at epilepsy-relevant CNS drug targets. We have recently reported that phytocannabinoids Δ^9^-tetrahydrocannabinolic acid (THCA) and CBGA inhibited the voltage-gated T-type calcium (Ca_v_3) channels (Mirlohi et al., 2021, 2022). Ca_v_3 channels, which comprise three subtypes, Ca_v_3.1, Ca_v_3.2, and Ca_v_3.3, are expressed in neuronal cells where they are activated by small membrane depolarisations and are considered neuronal pacemakers (Cribbs et al., 1998) Dysfunction of Ca_v_3 channels is associated with various medical conditions, including epilepsy, sleep disturbances and pain (Bourinet et al., 2005; Tringham et al., 2012). Inhibition of Ca_v_3.1 has been suggested as a novel target for Dravet syndrome as knockout of *Cacna1g*, which encodes Ca_v_3.1, diminished spontaneous seizures and improved survival of *Scn1a* ^+/*–*^ mice (Calhoun et al., 2017). Dicer small interfering (Dsi) RNA knockdown of *Cacna1h*, which encodes Ca_v_3.2, significantly shortened convulsions in the Genetic Absence Epilepsy Rats from Strasbourg (GAERS) model of absence seizures (Cain et al., 2018). Moreover, several anticonvulsant drugs, including ethosuximide and zonisamide, block T-type calcium channels (Baulac et al., 2012). CBD’s anticonvulsant mechanism of action might involve T-type channel inhibition because it inhibits peak Ca_v_3.1 and Ca_v_3.2 currents at clinically relevant concentrations (IC_50_ values = approximately 1 µM) (Ross et al., 2008). In the present study, we aimed to extend our prior research by examining whether a series of phytocannabinoids recently shown to exhibit anti-seizure properties inhibit T-type calcium channels.

## 2 Methods

### 2.1 Compounds

CBDA was purchased from THC Pharm GmbH (Frankfurt, Germany), and CBGV was purchased from Toronto Research Chemicals Inc. (Ontario, CAN). CBDVA, CBGVA, CBCA, and CBCVA were synthesised by the laboratory of Professor Michael Kassiou at the University of Sydney. CBCV was synthesised in-house. NNC 55-0396 and ML-186 were purchased from Cayman Chemical (Michigan, United States) and Enamine (New Jersey, United States) respectively. For patch clamp electrophysiology, CBGVA and CBDVA were purchased from Cerilliant Corporation (Texas, United States). Unless otherwise stated, all drugs were prepared as stock solutions in DMSO and diluted in HEPES-buffered low potassium Hanks Balanced Salt Solution (HBSS) with 0.01% bovine serum albumin (BSA, Sigma, Castle Hill, Australia). HBSS comprises (mM) NaCl 145, HEPES 22, Na_2_HPO_4_ 0.338, NaHCO_3_ 4.17, KH_2_PO_4_ 0.441, MgSO_4_ 0.407, MgCl_2_ 0.493, Glucose 5.56, CaCl_2_ 1.26; (pH7.4, osmolarity 315 ± 15 mOsmol). Final DMSO concentrations ranged between 0.1%–0.2%.

### 2.2 Cell culture

Human embryonic kidney 293 (HEK) Flp-In T-REx cells stably expressing T-type Ca_v_3.1, Ca_v_ 3.2, or Ca_v_ 3.3 channels were used (Bladen et al., 2021; Mirlohi et al., 2022). HEK 293 GPR55 cells were generously provided by Dr Julia Kargl (University of Graz, AUT) (Kargl et al., 2012). The cells were cultured in T75 flasks using Dulbecco’s modified Eagle’s medium (DMEM) (supplemented with 10% foetal bovine serum (FBS) and 1% penicillin-streptomycin (P/S; 100 U/ml), and incubated in a humidified atmosphere with 5% CO_2_ at 37°C. Blasticidin (10 μg/ml, Invivogen) and hygromycin (80 μg/ml) were used as the selection antibiotics for cells expressing Ca_v_3.1, Ca_v_ 3.2 or Ca_v_ 3.3, while G418 (400 μg/ml) was used for cells expressing GPR55. Cells were passaged at 80% confluency and used for assays at 90% confluency for up to 15 passages.

### 2.3 FLIPR calcium 5 assay

Calcium 5 dye (Molecular Devices, United States ) was used to investigate compound activities at T-type calcium channels using Flexstation III. HEK293 cells overexpressing Ca_v_3.1, Ca_v_ 3.2, and Ca_v_ 3.3 cells were resuspended in Leibovitz’s L-15 media containing 1% FBS, 1% P/S and 15 mM glucose. 90 µl of the resuspended cells (120,000 cells/well) were plated in a black-walled, 96-well microplate (Corning, NY, United States ) and incubated overnight in humidified air at 37°C. Channel expression was induced with tetracycline (2 μg/ml, Sigma-Aldrich) at plating. On the day of the assay, 90 µl of HBSS containing 5% Calcium dye (bulk kit) and 2.5 mM probenecid (Invitrogen, United States ) was added to each well and incubated for 1 h at 37°C. Change in fluorescence was measured in a FlexStation III plate reader every 2 s (ʎ_excitation_ = 485 nm, ʎ_emission_ = 525 nm) for the duration of the experiment at 37°C. After 2 min baseline recording, drugs (20 µL) were added and read for 5 min, followed by 10 mM CaCl_2_ (20 µl) and read for a further 3 min.

### 2.4 Alphascreen ERK phosphorylation assay

GPR55 cells were trypsinised and resuspended in Leibovitz’s (L-15) media containing 1% FBS, 1% P/S and 15 mM glucose. 110,000 cells/well were plated in a 96-well clear flat bottom CellBind™ microplate (Corning, NY, United States ) and incubated overnight for at least 21 h in humidified air at 37°C. L-15 was aspirated and replaced with 50 µl phenol red-free DMEM/F12 (Gibco, United States) and incubated in 5% CO_2_ at 37°C for 2 h. Each well was treated with 50 µl of drugs (prepared twice the final concentration) for 20 min at 37°C. Media was aspirated, 50 µL/well lysis buffer from the Alphascreen Surefire Kit was added, and the plate was placed on an orbital shaker at 500 rpm for 10 min. According to the manufacturer’s instructions, phosphorylated ERK was measured in 384-well white Proxiplate plates (Perkin Elmer, Australia). Briefly, 5 µl of lysate was incubated with 7 µl of Alphascreen reaction mixture in the dark for 3.5 h and read with a CLARIOstar reader (BMG Labtech) using standard AlphaScreen-compatible filters (excitation 680–40 nm, emission 570–100 nm).

### 2.5 Whole-cell voltage-clamp electrophysiology

All whole-cell voltage-clamp recordings from HEK293 Flp-In T-REx cells stably transfected with human Ca_v_3.1, Ca_v_ 3.2 and Ca_v_ 3.3 were performed at room temperature. At least 24 h prior to experiments, cells were detached from flasks using trypsin/EDTA and plated into 10 cm sterile tissue culture dishes containing 10 ml of supplemented DMEM and 10–15 glass coverslips (12 mm diameter, ProScitech, QLD, Australia). Culture dishes were then kept overnight in same conditions as flasks to allow cells to adhere to coverslips. They were then transferred to a 30°C/5% CO2 incubator to inhibit cell proliferation until ready to be used for electrophysiology experiments. At this stage, channel expression was induced with tetracycline (2 μg/ml, Sigma-Aldrich) 8–24 h prior to recording cells.

External recording solutions contained (in mM): 114 CsCl, 5 BaCl_2_, 1 MgCl_2_, 10 HEPES, 10 glucose, adjusted to pH 7.4 with CsOH. The internal patch pipette solution contained (in mM): 126.5 CsMeSO_4_, 2 MgCl_2_, 11 EGTA, 10 HEPES adjusted to pH 7.3 with CsOH. Internal solution was supplemented with 0.6 mM GTP and 2 mM ATP and mixed thoroughly just before use. Liquid junction potentials for the above solutions were calculated before experiments using pClamp 10 software and corrected during experiments. Compounds were prepared daily from 30 mM DMSO stocks and diluted into an external solution just before use. Compounds were then applied rapidly and locally to the cells using a custom-built gravity-driven micro-perfusion system (Feng et al., 2003). Initial vehicle experiments were performed to ensure that 0.1% DMSO did not affect current amplitudes or channel kinetics (data not shown), and all subsequent experiments contained 0.1% DMSO in control external solutions. Currents were elicited from a holding potential of -100 mV and were measured by conventional whole-cell patch clamp techniques using an Axopatch 200B amplifier in combination with Clampex 9.2 software. (Molecular Devices, Sunnyvale, CA). After establishing whole cell configuration, cellular capacitance was minimised using the amplifier’s built-in analogue compensation. Series resistance was kept to <10 MΩ and was compensated to at least 85% in all experiments. All data were digitised at 10 kHz with a Digidata 1,320 interface (Molecular Devices) and filtered at 1 kHz (8-pole Bessel filter). Raw and online leak-subtracted data were both collected simultaneously, P/N4 leak subtraction was performed using opposite polarity and after the protocol sweep.

For tonic inhibition of T-type currents, membrane potential was stepped from −100 mV to −30 mV for 200 ms and then allowed to recover for 12 s (1 sweep). A minimum of 10 sweeps were collected under vehicle control external perfusion to allow for control peak current to equilibrate. The drug was then continuously perfused, and sweeps were recorded until no further inhibition was seen (minimum of 3 sweeps with the same amplitude). In current-voltage relation studies, the membrane potential was held at −100 mV and cells were depolarised from −70 to 50 mV in 10 mV increments. For steady-state inactivation studies, a 3.6 s conditioning pre-pulse of various magnitudes (initial holding at −110 mV) was followed by a depolarising pulse to −30 mV. Individual sweeps were separated by 12 s to permit recovery from inactivation between conditioning pulses. The duration of the test pulse was typically 20 ms, and the current amplitude obtained from each test pulse was normalised to that observed at the holding potential of −100 mV.

### 2.6 Data analysis

Unless otherwise stated, five independent experiments were performed for each condition, with each experiment conducted in duplicate. For the calcium assay, change in fluorescence by 10 mM Ca in the presence of drugs was normalised to change in fluorescence caused by 10 mM Ca in the presence of vehicle only to allow for comparison. In the Alphascreen assay, results were normalised to EC_80_ of ML-186 after subtraction of basal ERK phosphorylation (presence of a vehicle and 0.2% DMSO) to allow for easy comparison between phytocannabinoid activities. We fitted all concentration-response curves to a 4-parameter sigmoidal inhibitory response curve in GraphPad Prism (Version 6 GraphPad Software Inc., CA, United States) to derive IC_50_ and Emax values. All statistical analyses were conducted with GraphPad Prism. Two-tailed, unpaired t-tests were used to compare two data points and one-way ANOVA for more than two data points with Tukey post-hoc analysis. *p*-values < 0.05 were considered statistically significant.

## 3 Results

### 3.1 Phytocannabinoids inhibit T-type Ca channels based on FLIPR assays

Our investigation of T-type channels began with a single-point screen, as described by Bladen et al. (2021). We screened the inhibitory effect of 10 µM phytocannabinoids on the change in fluorescence elicited by the addition of 10 mM Ca^2+^ in cells expressing Ca_v_3.1, Ca_v_3.2 or Ca_v_3.3 channels (Xie et al., 2007; Bladen et al., 2021). Compounds that displayed ≥80% inhibition of the 10 mM Ca^2+^ response were then further characterised with concentration-response curves (Bladen et al., 2021).

10 µM CBGVA and CBDVA inhibited calcium entry in cells expressing Ca_v_ 3.1 channels by 82 ± 6% and 80 ± 6%, respectively, which was similar in magnitude to the effect of 10 µM NNC550396 (82 ± 8%), an established non-selective T-type Ca channel inhibitor (McGivern, 2008). CBGV, CBCVA, CBCV, and CBCA showed <80% inhibition of calcium entry (Table 1).

**TABLE 1:** % inhibition of 10 mM Ca-induced fluorescence by phytocannabinoids (10 µM) at T-type calcium channels using FLIPR assay in HEK293 cells.

Compound	% Inhibition of Fluorescence		
	**Ca**_**v**_ **3.1**	**Ca**_**v**_ **3.2**	**Ca**_**v**_ **3.3**
NNC550396^*^	82 ± 8	97 ± 0.4	89 ± 2
CBCA	59 ± 9	28 ± 16	11 ± 10
CBCV	30 ± 10	-37 ± 21	1 ± 1
CBCVA	51 ± 8	62 ± 8	34 ± 12
CBDVA	80 ± 6	80 ± 6	8 ± 4
CBGV	51 ± 14	31 ± 9	6 ± 3
CBGVA	82 ± 7	91 ± 3	35 ± 11

^*^NNC550396 is a reference T-type calcium channel blocker. Compounds with ≥ 80% fluorescence inhibition were considered significant inhibitors and further characterised. Data presented are mean ± SEM, of five independent experiments conducted in duplicates.

CBGVA and CBDVA inhibited fluorescence in cells expressing Ca_v_3.2 by 91 ± 3% and 80 ± 6%, respectively. NNC550396 inhibited calcium entry by 89 ± 2% while CBGV, CBCVA and CBCA showed <80% inhibition. However, we observed that CBCV appeared to increase the influx of Ca^2+^ ions in cells expressing Ca_v_3.2 channels. At Ca_v_3.3, NNC550396 inhibited calcium influx by 89 ± 2%, but no tested phytocannabinoid showed ≥80% inhibition (Table 1).

**FIGURE 1:**
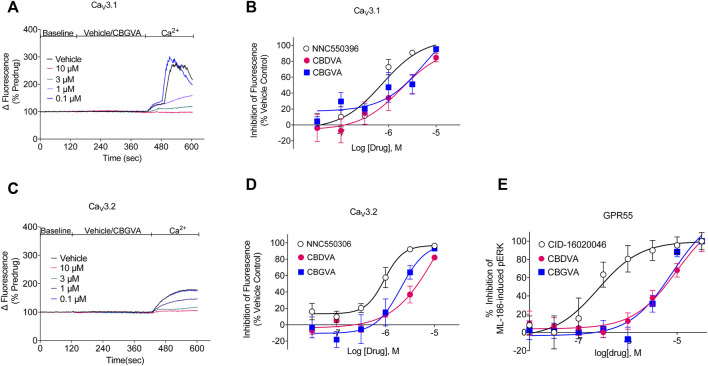
CBGVA and CBDVA inhibit Cav3.1 and Cav3.2 channels and GPR55 receptors. **(A)** A representative trace of the change in fluorescence caused by vehicle control and CBGVA in HEK 293 Ca_v_3.1 cells. After 2 min of baseline reading, vehicle or CBGVA were added for 5 min, followed by adding 10 mM Ca^2+^ and read for a further 3 min. **(B)** Concentration-response curves for NNC550396, CBGVA and CBDVA inhibition of Ca_v_3.1 response. **(C)** A representative trace of the change in fluorescence caused by vehicle control and CBGVA in HEK 293 Ca_v_3.2 cells. **(D)** Concentration-response curves for NNC550396, CBGVA and CBDVA inhibition of Ca_v_3.1 response. **(E)** Concentration-response curves of CBDVA and CBGVA against 0.45 µM ML-186 -induced ERK phosphorylation in HEK 293 cells expressing GPR55 receptors. All data presented are mean ± SEM of five independent experiments conducted in duplicates. Some data points do not have visible error bars due to low variability.

We next determined the concentration-response of CBGVA and CBDVA in cells expressing Ca_v_3.1 or Ca_v_3.2 channels. We did not determine the concentration-response of any phytocannabinoid on Ca_v_ 3.3 as no compound met the criterion for the single-point screen. CBGVA and CBDVA inhibited the change in fluorescence in cells expressing Ca_v_3.1 with IC_50_ values of 6 µM and 2 μM, respectively (Figures 1A,B). We observed similar potency in cells expressing Ca_v_3.2, with CBGVA and CBDVA having IC_50_ values of 2 µM and 11 μM, respectively (Figures 1C,D). Both phytocannabinoids were less potent than NNC550396, which exhibited IC_50_ values of 730 nM and 945 nM at Ca_v_3.1 and Ca_v_3.2 channels, respectively.

### 3.2 CBGVA and CBDVA block ML-186-induced ERK phosphorylation in GPR55 cells

We have recently shown that a major biosynthetic precursor molecule in cannabis, CBGA, displays anticonvulsant properties (Anderson et al., 2021b). CBGA inhibits T-type calcium channels but affects other targets, including antagonising GPR55. GPR55 is thought to contribute to the anticonvulsant mechanism of action of CBD (Kaplan et al., 2017). To determine whether CBGVA and CBDVA antagonise GPR55, we conducted concentration-response curves for CBGVA and CBDVA in the presence of the EC_80_ of ML-186 (Figure 1E). We found that CBGVA and CBDVA were low potency antagonists (IC_50_ = 8 µM and 10 μM, respectively) compared to the established GPR55 antagonist CID-16020046 (IC_50_ = 245 nM).

### 3.3 Single-cell patch-clamp electrophysiology shows CBGVA and CBDVA tonically inhibit Ca_v_3.1 currents

**FIGURE 2:**
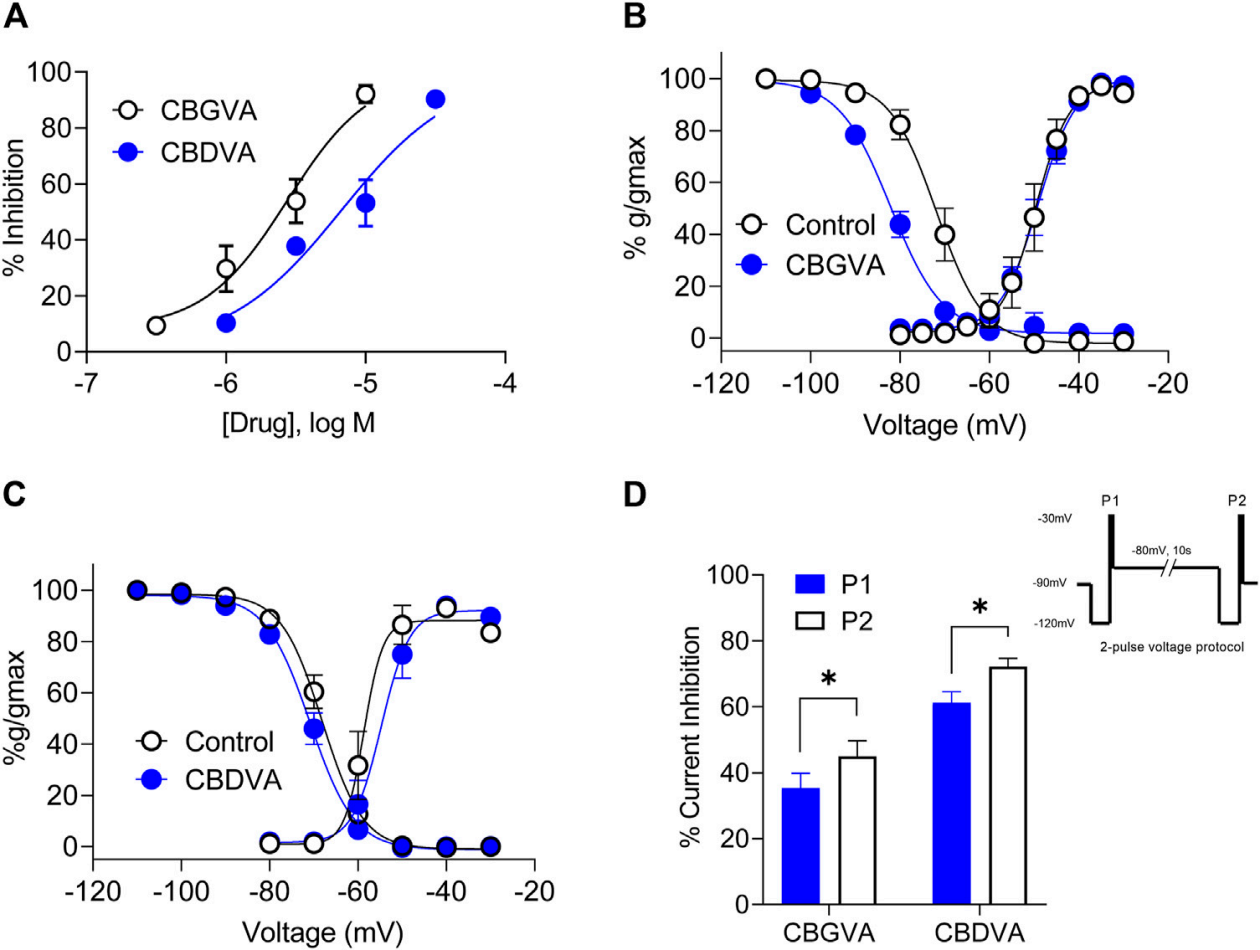
CBGVA and CBDVA inhibit Cav3.1 channel currents. **(A)** Concentration-response curve for CBGVA and CBDVA inhibition of Ca_v_3.1 channels. Whole-cell patch-clamp recordings were made from HEK T-rex Ca_v_3.1 channel currents evoked by stepping −100 mv to −30 mv every 12 s. Steady-state activation and inactivation curves of HEK Ca_v_3.1 in the absence (control) and presence of **(B)** CBGVA (5 µM) **(C)** CBDVA (5 µM). Data presented are mean ± SEM of 10–11 cells. **(D)** Ca_v_3.1 channel slow inactivated state was elicited by a 2-pulse voltage protocol (inset). This elicited a significant increase in peak current inhibition by CBGVA and CBDVA. Data presented are mean ± SEM of % inhibition of peak current of vehicle control for 9 cells. Statistical analysis was performed using a student T-test with * denoting *p* < 0.05. Some data points do not have visible error bars due to low variability.

Ca_v_3.1 is an emerging target of interest for treating intractable epilepsies, as it has been shown that genetic deletion of *Cacna1g*, which encodes Ca_v_3.1, reduces spontaneous seizure frequency in a mouse model of Dravet syndrome (Calhoun et al., 2017). Thus we explored further the inhibitory effects of CBGVA and CBDVA on Cav3.1 channels using single-cell electrophysiology. We measured tonic inhibition by repetitively stepping the cell membrane potential from −100 mV to −30 mV to elicit peak amplitude while superfusing concentrations of the tested phytocannabinoids. We found that CBGVA and CBDVA inhibited Ca_v_3.1 current amplitude in a concentration-dependent manner with IC_50_ values of 3 µM and 7 μM, respectively (Figure 2A).

### 3.4 The effect of CBGVA and CBDVA on activation and inactivation states of Ca_v_3.1 channels

We next investigated whether CBGVA and CBDVA affected Ca_v_3.1 channel gating by examining the effect of each compound (5 µM) on steady-state inactivation (SSI), the voltage dependence of activation and cumulative slow inactivation of Ca_v_3.1. We found that CBGVA significantly shifted SSI curves towards more hyperpolarised membrane potentials with a half-inactivation (V_0.5_) of −82.1 ± 0.8 mV; control −71.8 ± 0.9 mV. CBDVA had a negligible effect on steady-state inactivation (−70.9 ± 0.5 mV; control −68.1 ± 0.5 mV) (Figures 2B,C).

Both CBGVA (V_0.5_ value of −49.1 ± 0.5 mV; control −49.6 ± 0.9 mV) and CBDVA (V_0.5_ value of −54.7 ± 1.2 mV; control −58.6 ± 1.5 mV) did not significantly alter voltage dependence of Ca_v_3.1 activation (Figures 2B,C).

The effect of CBGVA and CBDVA (5 µM each) on slow inactivation was tested using a 2-pulse voltage protocol as described in Bladen *et al.,* (2021). Briefly, cells expressing Ca_v_3.1 were initially depolarised with a 200 ms test pulse from -120 mV to −30 mV to generate peak current (P1) and followed by a 10 s pre-conditioning pulse to −80 mV to reverse fast inactivation. Cells were further depolarised with another 200 ms test pulse from −120 to −30 mV (P2) (Figure 2 inset). We found that CBGVA inhibition of Ca_v_3.1 current significantly increased from 61 ± 3% to 72 ± 3%, and CBDVA inhibition increased from 35 ± 5% to 45 ± 5% (Figure 2D), suggesting that both compounds more significantly inhibit Ca_v_3.1 channels in the slow inactivated state than a fast inactivated state.

### 3.5 CBGVA and CBDVA inhibition of Ca_v_3.1 is frequency-dependent

**FIGURE 3:**
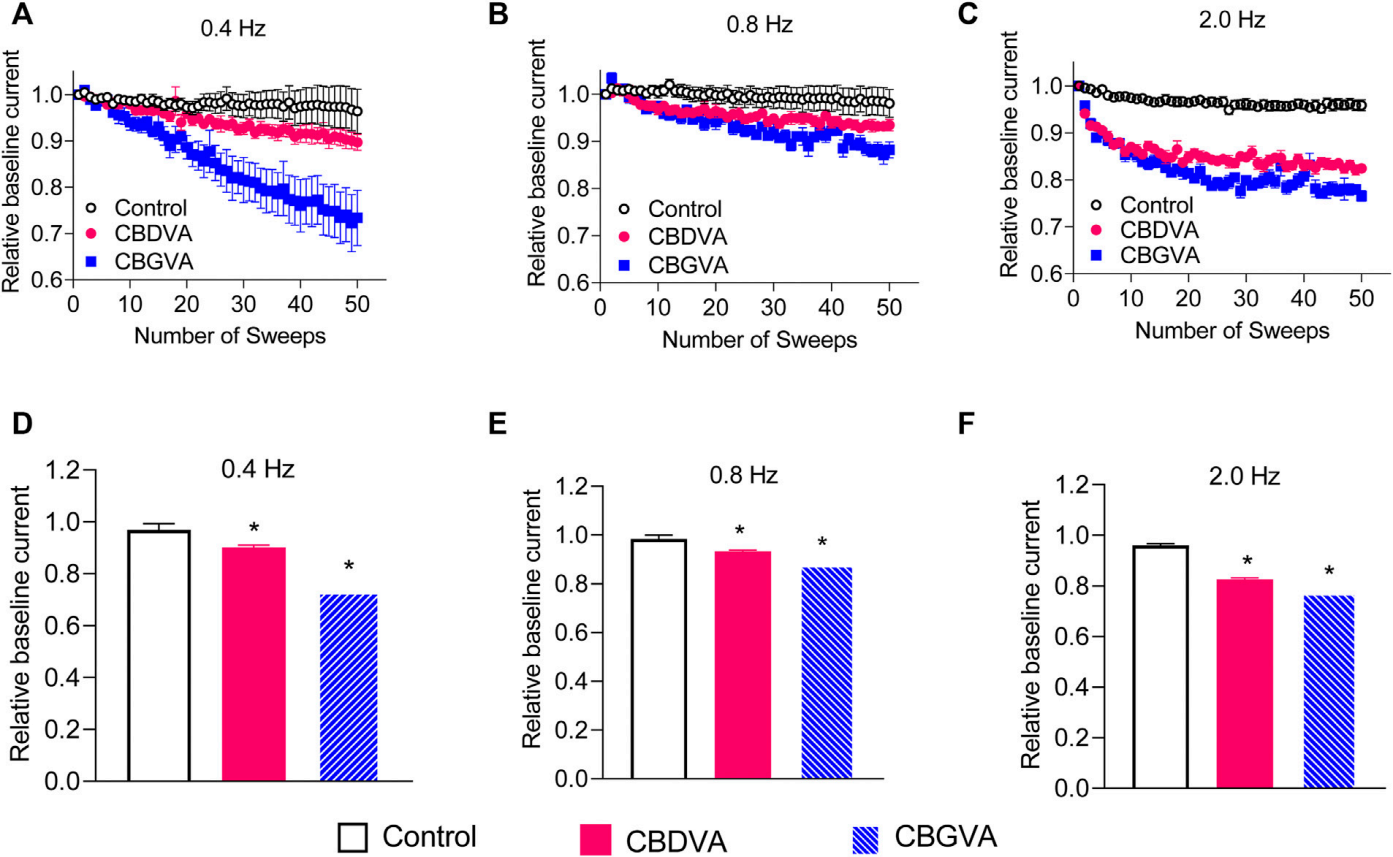
CBGVA and CBDVA block Cav3.1 channels in a frequency-dependent manner. In the absence (control) or presence of 5 µM CBGVA and CBDVA, cells were held at −100 mV and stepped at three different frequencies **(A)** 0.4 Hz **(B)** 0.8 Hz, **(C)** 2.0 Hz to −30 mV with intersweep holding potential of −100 mV. Data presented are mean ± SEM of peak currents of each sweep after normalisation to the average peak current of the baseline sweeps (first three sweeps) in 5–11 cells. Summary data of the relative current of CBDVA and CBGVA at **(D)** 0.4 Hz, **(E)** 0.8 Hz **(F)** 2.0 Hz. Data presented are mean ± SEM of the last three sweeps in 5–11 cells. Statistical analysis was performed using a student t-test with * denoting *p* < 0.05.

THC and CBD cause substantial frequency-dependent inhibition of Ca_v_3.2 and Na_v_1.8 channels, respectively (Ross et al., 2008; Bladen et al., 2021; Zhang and Bean, 2021). Therefore, we explored a possible use-dependent effect of CBGVA and CBDVA on Ca_v_3.1 action potentials delivered in 50 sweeps at frequencies of 0.4, 0.8 and 2 Hz. CBGVA caused a higher frequency-dependent reduction than CBDVA across all tested frequencies (Figures 3A–F). At the 50th action potential, 5 µM CBGVA reduced Ca_v_3.1 relative current to 73 ± 3%; 88 ± 1% and 77 ± 1% of that evoked by the first action potential during 0.4 Hz, 0.8 Hz and 2.0 Hz stimulation, respectively. 5 µM CBDVA also reduced Ca_v_3.1 current to 90 ± 1%; 93 ± 1%, and 83 ± 1% when stimulated at 0.4 Hz, 0.8 Hz and 2.0 Hz. CBGVA and CBDVA produce a modest use-dependent reduction of Ca_v_3.1 currents.

## 4 Discussion

We have recently reported that a series of acidic biosynthetic precursor molecules found in cannabis display anticonvulsant properties in the *Scn1a* ^+/−^ mouse model of Dravet syndrome, including CBGVA and CBDVA (Anderson et al., 2019; Anderson et al., 2021a; Anderson et al., 2021b). However, the molecular pharmacology of these acidic compounds is poorly understood. The present investigation provides evidence that these two anticonvulsant phytocannabinoids inhibit T-type calcium channels (Ca_v_3.1 and Ca_v_3.2) and GPR55 receptors at low micromolar concentrations. Given that Ca_v_3.1 is an emerging target for treating Dravet syndrome, we conducted more detailed patch-clamp electrophysiology studies to illuminate the biophysical properties of the inhibitory effects at this channel. We found that at 5 μM, CBGVA negatively shifted the half-inactivation voltage of Ca_v_3.1, while CBDVA did not. Both CBDVA and CBGVA did not significantly alter the half activation of Ca_v_3.1 channels. Interestingly, both compounds displayed use-dependent blockade of Ca_v_3.1 current and higher inhibition of Ca_v_3.1 current in a slow than a fast inactivated state.

Our findings that the minor phytocannabinoids CBDVA and CBGVA inhibit T-type calcium channels extend upon research showing that phytocannabinoids and synthetic cannabinoids inhibit these channels (Ross et al., 2008; Mirlohi et al., 2021, 2022). We recently reported that the structurally related phytocannabinoid CBGA, which is also anticonvulsant, inhibited Ca_v_3.1 and Ca_v_3.2 channels (Mirlohi et al., 2022). Here CBDVA and CBGVA displayed lower potency than CBGA using an identical fluorescent reporter assay. Moreover, CBGVA and CBDVA affected the biophysical properties of the channel distinctly to CBGA. At concentrations similar to the EC_50_ of tonic inhibition, CBGA caused a significant negative shift of both fast inactivation and activation of Ca_v_3.1 current (Mirlohi et al., 2022). Whereas in this study, CBGVA negatively shifted fast inactivation but not activation, and CBDVA did not affect either state. The preferential shift of the fast inactivation curve in Ca_v_3.1 channels by CBGVA is similar to CBD, CBG and CBDV at Ca_v_3.1 channels (Ross et al., 2008; Mirlohi et al., 2022). Hyperpolarising shifts of steady-state fast inactivation suggest preferential binding of CBGVA to the inactivated state of the Ca_v_3.1 channels (Freeze et al., 2006; Eckle and Todorovic, 2010). Future studies should investigate the effect of CBGVA and similar phytocannabinoids on the rate of Ca_v_3.1 recovery from inactivation because drugs that stabilise inactive states of channels are often simultaneously slowing the recovery from inactivation due to slow drug dissociation (Burgess et al., 2002; Talavera et al., 2004; Ghovanloo et al., 2022).

In this study, CBGVA and CBDVA more effectively inhibited Ca_v_3.1 channels in a slow inactivation state, similar to CBD (Bladen et al., 2021). CBD is also reported to preferentially bind the slow inactivated state of Na_v_1.8 channels (Zhang and Bean, 2021). Up to 90% of Na_v_1.8 channels can be in a slow inactivated state at depolarised potentials (Tripathi et al., 2006), and a slow inactivated state of Ca_v_3.1 can reduce calcium currents by up to 50% (Hering et al., 2004). Given that T-type calcium channels critically determine the threshold of Na^+^ channel all or none firing, it can be posited that CBD’s preferential binding to slow inactivated states may be a major contributory mechanism that reduces neuronal excitability in epilepsy. This could also apply to CBDVA and CBGVA’s anticonvulsant properties, but it is unclear whether they inhibit Na^+^ channels, which could be addressed in future research.

Phytocannabinoids tend to be multi-modal and affect numerous drug targets, which may contribute to their efficacy as potential therapeutic agents (Brodie et al., 2015). To advance knowledge on the molecular pharmacology of CBDVA and CBGVA, we also demonstrated here that these compounds antagonise GPR55. GPR55 is thought to contribute to the anticonvulsant effects of CBD (Gray and Whalley, 2020; Kaplan et al., 2017), and a growing list of anticonvulsant phytocannabinoids such as CBGA and CBDV share this property (Anavi-Goffer et al., 2012; Anderson et al., 2021b). Future studies in preclinical epilepsy models are needed to assess whether GPR55 and T-type calcium channels are necessary for the anticonvulsant effect of these phytocannabinoids. It would also be interesting to explore the effects of these phytocannabinoids in other disease models in which T-type channels and GPR55 play a role, such as in models of insomnia, pain, and gastrointestinal disorders (Harding and Zamponi, 2022). Given the promiscuity of the phytocannabinoids, additional cellular pharmacology studies are needed to address whether CBGVA and CBDVA affect other phytocannabinoid targets such as cannabinoid receptors and transient receptor potential (TRP) channels. Notably, recent studies reported that CBDVA did not alter excitatory neurotransmission in autaptic hippocampal neurons, which predominantly express cannabinoid CB1 receptors (Straiker et al., 2021). Further, CBDVA did not alter calcium responses in dorsal root ganglion neurons (which express a mixed population of TRP channels). CBDVA has been shown to inhibit the endocannabinoid synthetic enzyme diacylglycerol lipase at a relatively high concentration (de Petrocellis et al., 2011).

An important question arises as to whether the effects we observed here are relevant to the levels of exposure associated with the anticonvulsant effects of CBDVA and CBGVA in preclinical models (Anderson et al., 2021b). Peak plasma concentrations of CBGVA and CBDVA at anticonvulsant doses in mice are approximately 1,200 µM and 239 μM, respectively (Anderson et al., 2021b). The brain-plasma ratios of CBGVA and CBDVA are 4% and 2%, respectively (Anderson et al., 2019). Thus, anticonvulsant doses would be expected to achieve brain concentrations of 48 μM and 5 µM for CBGVA and CBDVA, respectively. Therefore, the effects of CBGVA and CBDVA on GPR55 receptors and T-type calcium channels might be relevant to the anticonvulsant effects of these compounds. However, the limited brain uptake of these molecules would need to be surmounted for these compounds to be advanced as therapeutics for CNS disorders.

It is unlikely that the effects observed in this study will be experienced after using medicinal cannabis products because these products generally contain negligible concentrations of these phytocannabinoids (Hanuš et al., 2016; Suraev et al., 2018). However, it is noteworthy that CBDVA was the most abundant phytocannabinoid found in a newly characterised Chinese accession (G-309) of a hemp form of cannabis with low THC content (Muscarà et al., 2021). This highlights that such phytocannabinoids might not necessarily be “minor” in some cannabis strains. Moreover, a new enzymatic bioreactor technology can synthesise CBGVA and CBDVA at scale with high purity (Valliere et al., 2019). This reiterates the significance of investigating the activities of minor phytocannabinoids because they could be used in new cannabinoid formulations.

## 5 Conclusion

Here we report that the understudied minor phytocannabinoids CBDVA and CBGVA, which are biosynthetic precursor molecules found in the cannabis plant, inhibit both T-type calcium channels and GPR55 receptors *in vitro*. Our data suggest that these compounds could be further explored for therapeutic potential in disease states which involve these channels or receptors, such as epilepsy, insomnia, pain and gastrointestinal disorders.

## Data Availability

The original contributions presented in the study are included in the article/Supplementary Material, further inquiries can be directed to the corresponding authors.
